# How I do it: dual operation channels percutaneous endoscopic far-lateral transforaminal lumbar interbody fusion

**DOI:** 10.1007/s00701-024-05946-x

**Published:** 2024-02-02

**Authors:** Qingqing Xiao, Ji Wu, Fuming Chu, Yue Li

**Affiliations:** Neck-Shoulder and Lumbocrural Pain Division 1, Sichuan Province Orthopedic Hospital, Chengdu, 610041 China

**Keywords:** Far-lateral, Transforaminal, Dual operation channels, Percutaneous endoscopic, Lumbar interbody fusion

## Abstract

**Background:**

The current surgical procedure of interbody fusion in the lumbar spine has several limitations including low efficiency, potential endplate damage, overdose radiation exposure, and failure of fusion.

**Methods:**

Through the endoscopic operating channel, we efficiently removed the superior and inferior articular processes and decompressed the ligamentum flavum. Another operating channel was established under endoscopic monitoring to excise the annulus fibrosus, remove the cartilaginous endplate using open instruments, perform interbody bone grafting, and place a non-expandable polyetheretherketone open surgical fusion cage.

**Conclusion:**

Lumbar interbody fusion was performed successfully using a far-lateral transforaminal approach combined with dual operation channels of percutaneous endoscopic-assisted technique.

**Supplementary Information:**

The online version contains supplementary material available at 10.1007/s00701-024-05946-x.

## Relevant surgical anatomy

Since the initial report in 1996 [1], there have been numerous studies documenting the advancements in surgical techniques and favorable clinical outcomes of lumbar interbody fusion assisted by spinal endoscopy [2–7]. Compared to other minimally invasive procedures using air medium, such as microscopic-assisted or tubular-assisted lumbar interbody fusion, endoscopic-assisted lumbar interbody fusion offers significant advantages in terms of surgical visualization, reduced intraoperative bleeding, minimized muscle and facet damage, and accelerated postoperative recovery. Percutaneous endoscopic lumbar interbody fusion could be performed through the intervertebral foramen or the interlaminar space [8, 9]. There are several limitations associated with the excision of the cartilaginous endplate, intra-disc bone grafting, and placement of interbody fusion cage after decompression. The commonly used fusion cages include expandable cylindrical fusion cages and non-expandable polyetheretherketone bullet-shaped fusion cages. However, these devices have limited capacity for bone grafting within the disc space, leading to the risk of implant subsidence and failure of intervertebral fusion. How to ensure safe and efficient decompression, select larger fusion cages, and keep visible during percutaneous endoscopic fusion surgery is challenging and requires improvement.

## Description of the technique

We attempted a minimally invasive transforaminal endoscopic-assisted lumbar interbody fusion using a dual-channel approach through the far-lateral intervertebral foramen. Under endoscopic guidance, we performed decompression, including resection of the facet joints and ligamentum flavum, and created an additional working channel for annulus fibrosus removal. Through the dual operation channels, we achieved intervertebral disc removal, extensive endplate preparation, and insertion of a non-expandable polyetheretherketone fusion cage typically used in open surgery. The procedure was successfully completed with direct visualization under endoscopy throughout the surgery, ensuring an unobstructed surgical field. Adequate bone grafting was performed in the disc space and inside the fusion cage.

## Surgical instruments

**Fig. 1:**
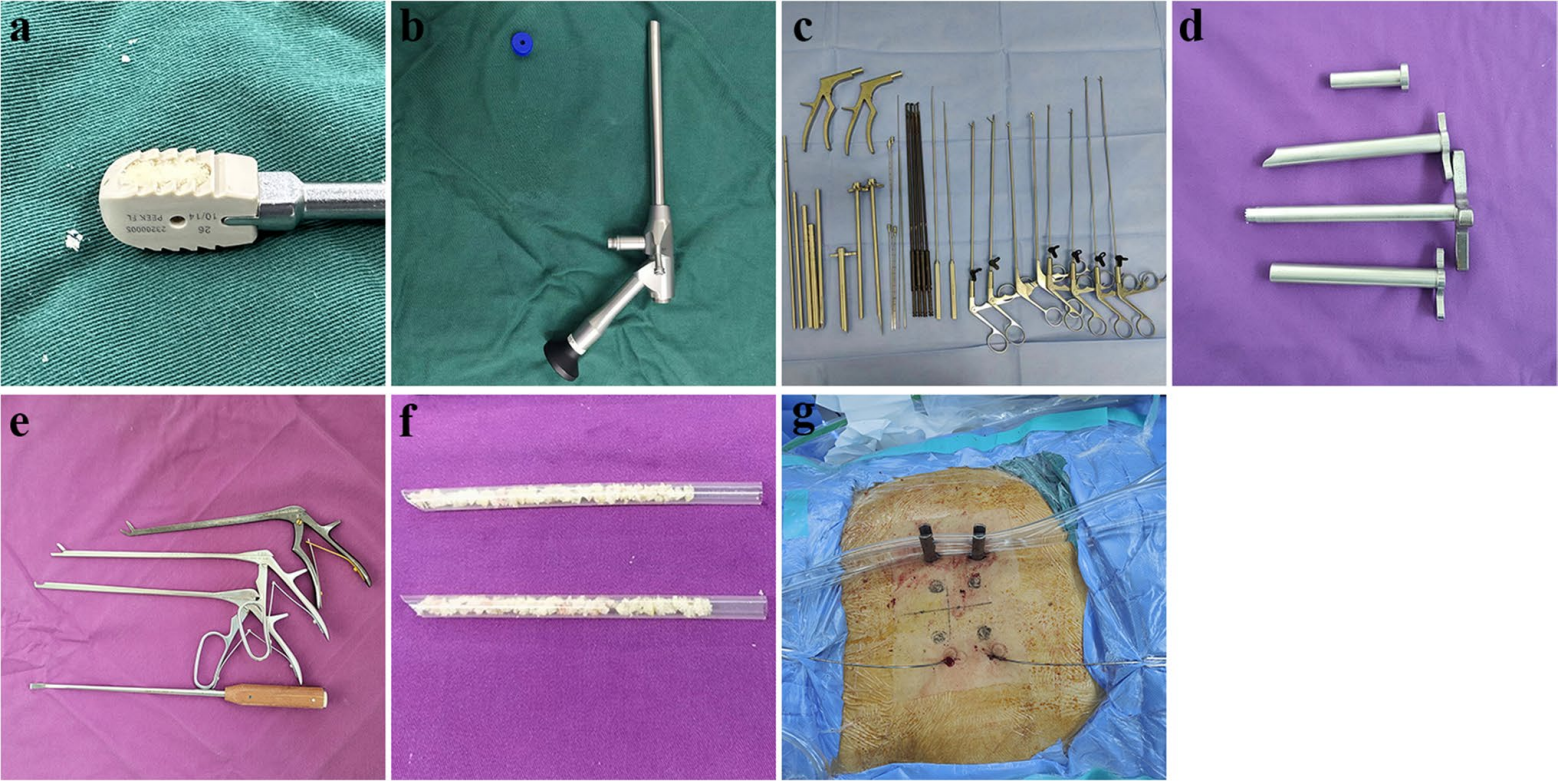
Fusion cage, special instruments used for lumbar interbody fusion technique and preoperative preparations for fusion surgery. **a** Non-expandable polyetheretherketone open surgical fusion cage. **b–d** LUSTA large-channel endoscope and instruments, with an outer visual circular saw. **e** Open surgical instruments including Kerrison bone rongeurs, upward-mobilizing nucleus pulposus forceps, straight nucleus pulposus forceps, and angled bone knife. **f** Autogenous bone, allogenic bone, and bone grafting tools. **g** Preoperative preparation requires the placement of guide wires, followed by the insertion of two pedicle screws on the contralateral side

A non-expandable polyetheretherketone fusion cage for open surgery (Fule Technology, Beijing, China) (Fig. 1a). The fusion cage has a width of 10 mm, a length of 22–32 mm, and a height of 8–14 mm. The LUSTA large-channel endoscope, with a 15° angle, has an outer diameter of 10 mm, an inner diameter of 7.1 mm, and a length of 139 mm. The system (Spinendos, Germany) includes intraoperative tools, the external ring saw, and the working cannula (Fig. 1b–d). Conventional instruments for open spine surgery, such as Kerrison rongeurs, up-lifting nucleus rongeurs, straight nucleus rongeurs, and specifically designed angled bone knives (Fig. 1e), are also used. Additionally, disposable radiofrequency plasma knife heads are used (BONSS Medical Technology, Taizhou, China).

## Position and creation of one portal

After inserting four guide wires, the incision is designed. Two 1.5-cm incisions are made along the opposite side around the guide wires. Then, two pedicle screws are first inserted. For the decompression side, percutaneous endoscopic decompression surgery is performed by making an approximately 1.2 cm incision at a distance of 2.0 cm from the outer edge of the pedicle. A working cannula and an endoscope are inserted through the incision (Fig. 1g).

## Facetectomy and flavectomy

**Fig. 2:**
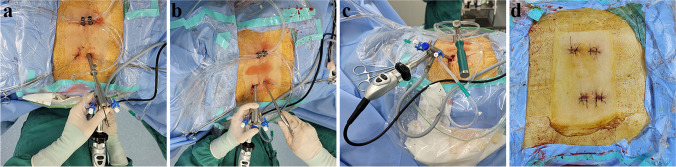
Overview of the dual operation channels percutaneous endoscopic lumbar interbody fusion and surgical incision. **a** Percutaneous endoscopic operating channel exposure. **b** Exposure after establishing an additional operating channel. **c** Both operating channels can accommodate instruments for manipulation. **d** Postoperative incision

**Fig. 3:**
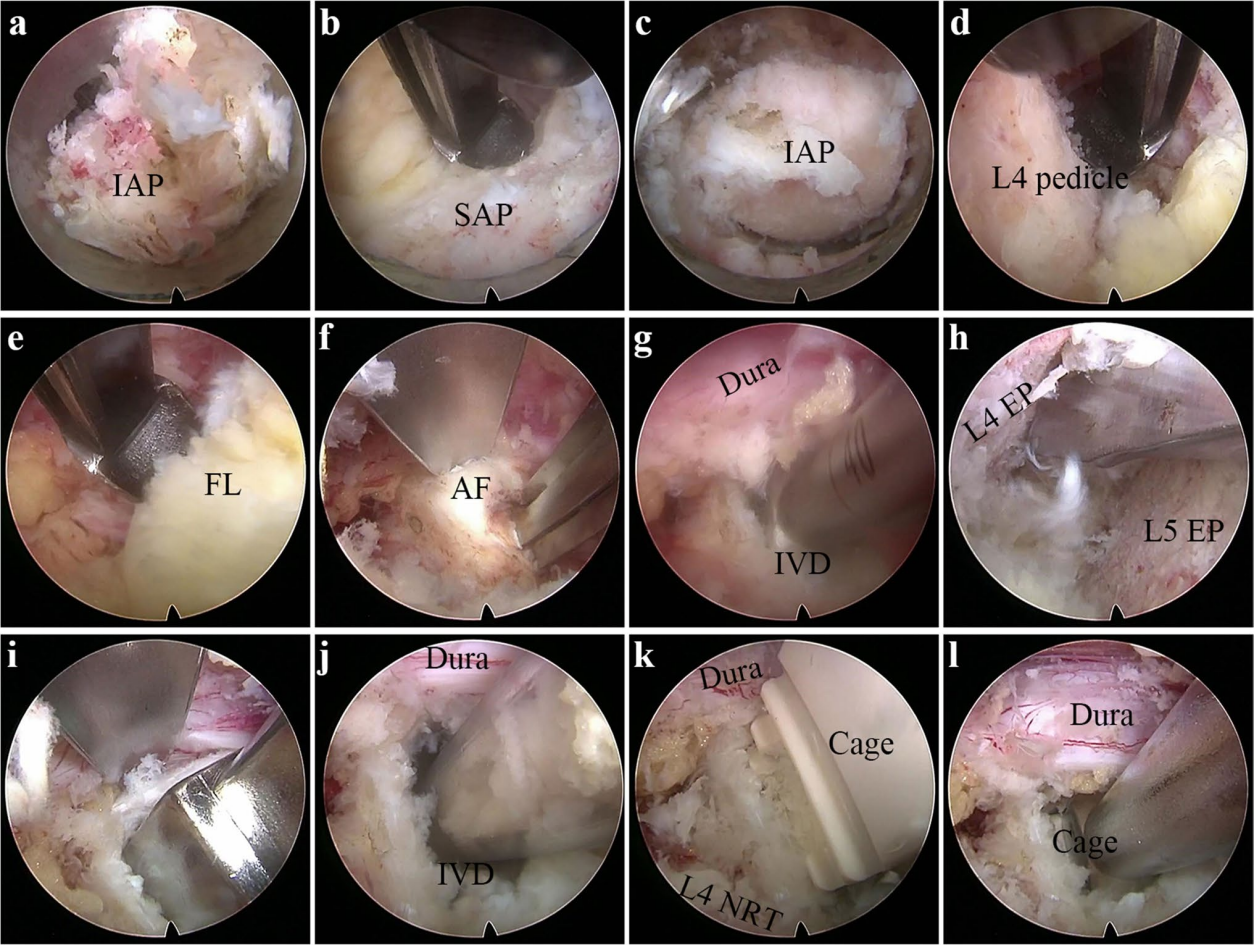
Intraoperative photographs, surgical technique steps. **a** Resection of the tip of the L4 inferior articular process. **b** Decompression along the inner, upper, and outer edges of the L5 vertebral arch. **c** Resection of the L4 inferior articular process under microscopic guidance. **d** Decompression along the inner, lower, and outer edges of the L4 vertebral arch. **e** Resection of the ligamentum flavum within the spinal canal and intervertebral foramen. **f** Sharp excision of the annulus fibrosus after the endoscope-assisted retraction of the dural sac. **g** Insertion of a chisel to manipulate the intervertebral disc; **h** Use of an angled bone knife to scrape the cartilaginous endplates. **i** Trial reduction after the endoscope-assisted retraction of the dural sac. **j** Bone grafting within the intervertebral space under full endoscopic visualization. **k** Placement of an interbody fusion cage. **l** Adequate positioning of the interbody fusion cage. Orientation of the field of view under full endoscope: left, cephalad side; right, caudal side; upper, medial side (left side); and lower, lateral side. SAP superior articular process, IAP inferior articular process, FL indicates flavum ligament, AF, annulus fibrosis, EP endplate, and NRT nerve root. IVD intervertebral disc

The L4 inferior articular process and L4 superior articular process are exposed (Fig. 2a). The ring saw is used to remove a portion of the tip of the L4 inferior articular process (Fig. 3a). This exposes the L5 lamina and the medial edge of the superior articular process. The L5 lamina is partially removed using a laminar rongeur, decompressing it up to the inner edge of the L5. Part of the superior articular process is removed along the inner edge of the L5 pedicle using the laminar rongeur, extending it to the outer edge of the L5 pedicle (Fig. 3b). The ring saw is used to remove the L4 inferior articular process (Fig. 3c), and the L4 lamina is partially removed using a laminar rongeur. By touching the inner edge of the L4 pedicle with the laminar rongeur, the residual inferior articular process is removed along the lower edge of the L4 pedicle to the outer edge of the pedicle (Fig. 3d). The left-sided ligamentum flavum is removed using the laminar rongeur, and adequate hemostasis is ensured (Fig. 3e).

## Discectomy, cage implantation, and endplate preparation

Under the guidance of percutaneous endoscopic monitoring, establish an operating channel by making a surgical incision with a diameter of 1.5 cm, approximately 2.0 cm away from the outer edge of the L4 vertebral arch, while ensuring a minimum distance of 3.0 cm from the proximal and distal ends of the percutaneous endoscopic channel (Fig. 2b). After inserting the intradiscal cannula through the endoscope, the tip of the cannula can be used to gently retract the dural sac, use a long-handled scalpel to sharply excise the fibrous ring (Fig. 3f), a curette to treat the intervertebral space (Fig. 3g), and forceps to remove the intraspinal nucleus pulposus and protruding nucleus pulposus within the disc space. Use an angled bone knife to prepare the upper and lower endplates of the vertebral bodies until the bony surfaces have sufficient bleeding as a preparation for the graft bed (Fig. 3h). After using the tip of the intradiscal cannula to gently retract the dural sac during percutaneous endoscopic procedures, the trial insertion can be performed (Fig. 3i), select an appropriately sized intervertebral fusion device. Fill the intervertebral space with autogenous bone and allograft bone through a plastic cannula (Figs. 1f and 3j). Under endoscopic visualization, insert the intervertebral fusion cage into the disc space (Fig. 3k), ensuring that there is no compression on the traversing root and exiting root (Fig. 3l), gradually achieving a satisfactory depth under C-arm fluoroscopy. Achieve adequate hemostasis and ensure there are no free bone fragments around the nerves (Fig. 2c), then place a gelatin sponge behind the disc space.

## Pedicle screw fixation

**Fig. 4:**
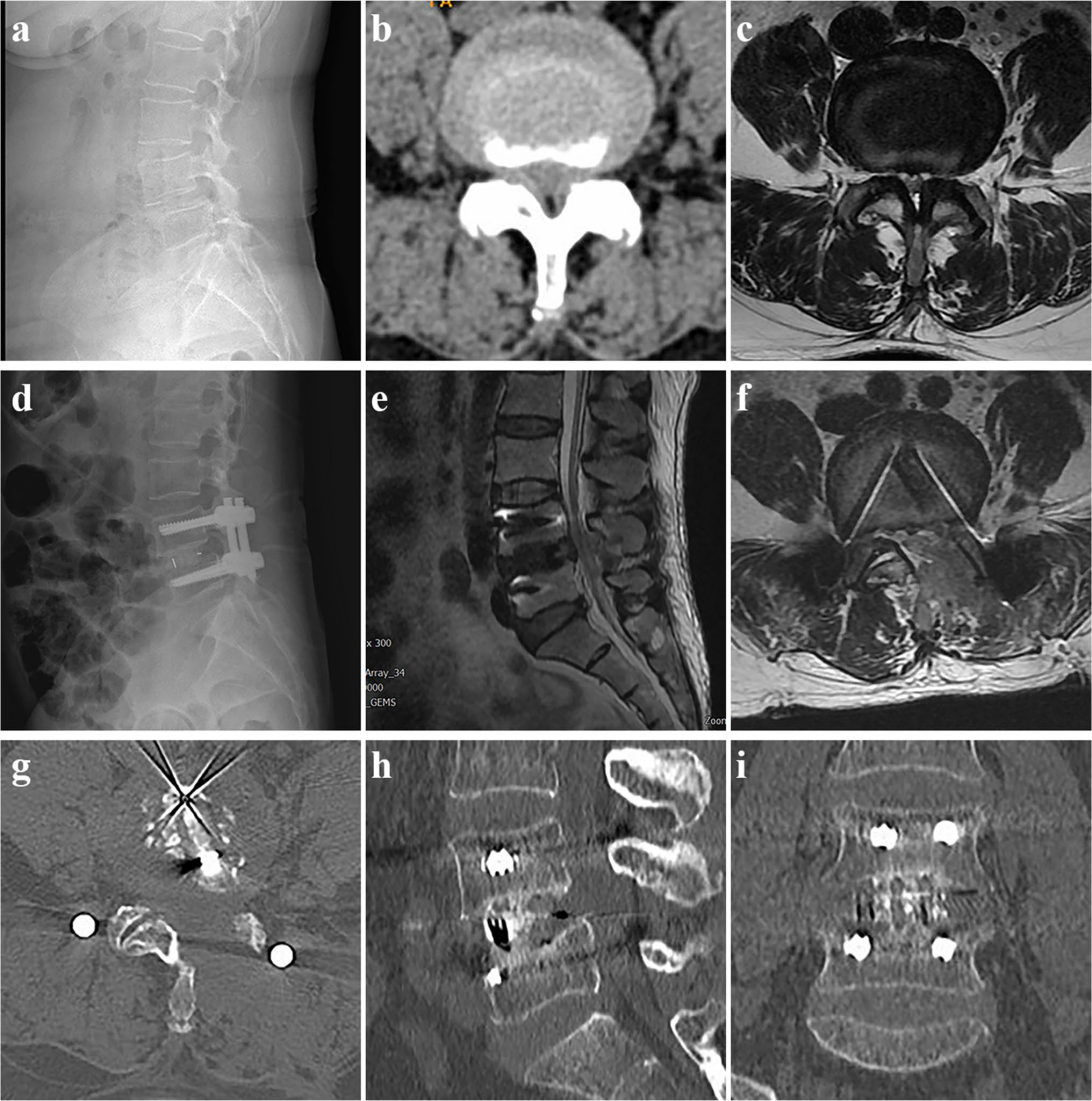
A 58-year-old female with grade 1 spondylolisthesis at L4. **a** The X-ray indicates grade 1 spondylolisthesis at L4. **b**–**c** There is spinal stenosis at the L4–5 level and narrowing of the right neural foramen. **d** Postoperative X-ray shows a complete reduction of the spondylolisthesis. **e**–**f** The postoperative follow-up MRI suggests sufficient decompression of the spinal canal and left lateral recess. **g**–**i** Postoperative CT scan showed complete interbody fusion of L4–5 at 6 months after surgery

Place two pedicle screws through the original incision on the decompression side and insert a pre-bent connecting rod. (If there is associated spondylolisthesis, place additional screws to realign the slipped vertebra and use the pre-bent connecting rod to reduce the slippage). Close the surgical incision with sutures, do not place a plasma drainage tube (Figs. 2d and 4).

## Indications

(1) Lumbar degenerative disc disease; (2) discogenic lower back pain caused by annular tear or vertebral endplate inflammation; (3) segmental instability of the lumbar spine; (4) grades 1–2 degenerative spondylolisthesis of the lumbar spine; (5) lumbar spondylolysis and grades 1–2 spondylolisthesis of the lumbar spine; and (6) iatrogenic lumbar instability caused by surgical disruption of stable structures during lumbar spine surgery.

## Limitations

This surgical approach shows similar limitations that exist in percutaneous endoscopic lumbar interbody fusion and unilateral biportal endoscopy endoscopic lumbar interbody fusion; it is also hard to observe the traversing nerve roots and exiting nerve roots in the same view when inserting the fusion cage. The endoscope needs to be rotated to change the perspective for observation while inserting the interbody fusion cage to prevent damage to the traversing nerve roots and exiting nerve roots. The operator should be proficient in both full endoscopic technique and unilateral biportal endoscopy technique.

## How to avoid complications

Under endoscopy, it is important to achieve sufficient hemostasis and avoid operating in cases of unclear vision. When using a ring saw for osteotomy, attention should be paid to the osteotomy plane to prevent injury to the superior vertebral body and pedicle. When using a vertebral plate bone clamp to identify the inner edge of the superior vertebral body and pedicle, the plate should be bitten off from the tail end to the head end, and after confirming the inner edge of the pedicle, decompression should be performed downward to the outer edge of the pedicle to reduce the risk of exiting nerve root injury. During the removal of the fibrous ring, an endoscopic working channel can be used to guide the retractor to elevate the dural sac and traverse the nerve root, thereby widening the width of the Kambin’s triangle and avoiding damage to the dural sac and traversing nerve roots, without the need for additional incisions. When inserting the interbody fusion device, it should be done slowly while observing that the traversing and exiting nerve roots are not compressed. It is recommended to place the fusion cage in the anterior part of the intervertebral space to avoid the device retracting into the intervertebral space during the later stage.

## Specific perioperative considerations

According to the preoperative bone density examination results, patients with severe osteoporosis may require cement augmentation with bone cement screws. Prior to surgery, measurements of the anterior–posterior diameter and left–right diameter of the vertebral body should be taken under CT bone three-dimensional reconstruction to select the appropriate size of the interbody fusion cage.

## Specific information to give to the patient about the surgery and potential risks

Although the bilateral transforaminal percutaneous endoscopic lumbar interbody fusion procedure offers the advantages of minimally invasive surgery, when using the LUSTA large-channel endoscope, there may be a risk of obstruction by the endoscope cannula during the placement of the interbody fusion cage if the distance between the two operative channels is less than 3 cm. Therefore, it is important to pay attention to the spacing between the two operative channels when creating an additional operative channel. If there is not enough space between the two operative channels, an alternative option is to switch to a unilateral biportal endoscopy for monitoring the placement of the interbody fusion cage. Currently, there is no literature reporting long-term clinical and radiological outcomes of endoscopic lumbar interbody fusion procedures [10].

## Supplementary Information

Below is the link to the electronic supplementary material.

Supplementary file1 (MP4 38201 KB)

## Data Availability

Available on request.
